# Factors associated with the number and size of renal angiomyolipomas in sporadic angiomyolipoma (sAML): a study of adult patients with sAML managed in a Dutch tertiary referral center

**DOI:** 10.1007/s11255-017-1766-9

**Published:** 2018-01-15

**Authors:** J. L. H. Ruud Bosch, Francis Vekeman, Mei Sheng Duh, Maureen Neary, Matthew Magestro, Jonathan Fortier, Paul Karner, Raluca Ionescu-Ittu, Bernard A. Zonnenberg

**Affiliations:** 1 Department of Urology, University Medical Center Utrecht, Heidelberglaan 100, 3584 CX Utrecht, Netherlands; 2 Groupe d’analyse, Ltée, 1000 De La Gauchetiere West, Suite 1200, Montréal, QC H3B 4W5 Canada; 3 Analysis Group, Inc., 111 Huntington Avenue 14th Floor, Boston, MA 02199 USA; 4 Novartis Pharmaceuticals Corporation, One Health Plaza, East Hanover, NJ 07936 USA; 5 Department of Internal Medicine, University Medical Center Utrecht, Heidelberglaan 100, 3584 CX Utrecht, Netherlands

**Keywords:** Angiomyolipoma, Treatment, Disease monitoring, Kidney function

## Abstract

**Purpose:**

To describe the patient characteristics, treatments, disease monitoring, and kidney function of patients with sporadic angiomyolipoma (sAML), stratified by the number and size of renal angiomyolipomas (AMLs).

**Methods:**

Single-center retrospective analysis of patients with sAML treated from 1990 to 2015 in a dedicated clinic for inheritable tumor syndromes in a tertiary referral center from the Netherlands. Patients’ first AML assessment at the clinic was defined as the index date. Patient characteristics were measured at the index date. Treatments, disease monitoring, and kidney function were measured post-index date.

**Results:**

The study sample included 53 patients followed for a total of 184.6 patient-years. At the index date, the largest AML was ≥ 3.5 cm for 26 patients and < 3.5 cm for 27 patients (including six patients with five or more AMLs of < 3.5 cm). As compared to patients with AMLs < 3.5 cm, patients with largest AML ≥ 3.5 cm had higher frequency of pre-index bleeding episodes (31 vs. 4%), pre-index hypertension (35 vs. 15%), post-index nephrectomy (19 vs. 4%), post-index embolization (8 vs. 0%), and post-index renal scans (1.14 vs. 0.74 scans/year). Kidney impairment was especially pronounced in young adults with AML ≥ 3.5 cm. On average, patients with sAML developed chronic kidney disease stage two earlier than the general Dutch population (age 42 vs. 55 years), but later than the patients with tuberous sclerosis complex (35 years).

**Conclusions:**

Patients with sAML, especially those with larger AMLs, have high disease burden.

**Electronic supplementary material:**

The online version of this article (10.1007/s11255-017-1766-9) contains supplementary material, which is available to authorized users.

## Introduction

Renal angiomyolipoma (AML) accounts for 2–6% of all kidney tumors [1]. A retrospective study of 61,389 patients without tuberous sclerosis complex (TSC) who underwent abdominal ultrasounds during routine care found an AML prevalence of 0.60% in females and 0.28% in males [2]. AML usually develops sporadically (sAML), but in up to 20% of the cases it may be associated with genetic disorders such as TSC [3]. sAML has later age onset, higher prevalence in women, and generally grows more slowly than TSC-related AML (TSC-AML) [4–6]. The literature on sAMLs is mostly based on small case series [6–9], and little is known about the natural history of sAML and patterns of treatment in clinical studies. Previous studies have shown that AML tumors grow at an average rate of 0.021–0.19 cm/year [6, 10, 11], leading sometimes to life-threatening complications such as aneurysmal vessel rupture or retroperitoneal hemorrhage [12, 13].

Embolization and nephrectomy are interventions recommended to patients at higher risk of potentially life-threatening bleeding complications and flank pain due to large AMLs [14]. While these invasive interventions are reliable in preventing or treating active bleeding and for improving flank pain symptoms, they may affect kidney function by damaging the renal tissue [15]. Active surveillance is the most common sAML disease management option not involving an invasive kidney intervention, although mammalian target of rapamycin inhibitors (mTORi) may be used off-label in some patients with sAML, based on their mechanism of action in TSC-related AML [16].

This study describes patient characteristics, treatments, disease monitoring, and kidney function of patients with sporadic angiomyolipoma (sAML), stratified by the number and size of renal angiomyolipomas.

## Methods

The study used a single-center retrospective chart-review study design. Data were collected from the medical charts of patients managed at the University Medical Center Utrecht (UMCU) from January 1990 to April 2015. The UMCU is a major specialty center in the Netherlands for patients with kidney tumors and a tertiary center with dedicated clinic for inheritable tumor syndromes. Most patients receive their first kidney tumor diagnosis at UMCU after an ultrasound or computerized tomography (CT) scan referral, but previously diagnosed patients may also be referred for second opinion or may present with acute bleeding in the emergency department.

Patients with an imaging or pathological diagnosis of kidney tumor in the UMCU medical records were reviewed and considered eligible if (1) the tumor was confirmed as sAML and (2) were never diagnosed with malignant renal tumors, TSC, or tuberculosis. (The latter were excluded because of possible misclassification in the hospital information system.) Pulmonary lymphangioleiomyomatosis [LAM] was not an exclusion criterion. Demographic and clinical information was extracted from the medical charts of eligible patients. The date of the first AML assessment at the UMCU was defined as the index date. Treatments, disease monitoring, and kidney function were measured from the index date to the date of death, date of the last follow-up at the UMCU, or date of data collection (April 2015), whichever came first (follow-up period). Pre-index information was collected from both the patient medical history taken at the time of the first AML assessment and, where applicable, from the patient’s hospital records prior to the index date. All patients were seen, tested, and treated as part of the regular care at UMCU. The study was approved by the UMCU Institutional Review Board (study METC 14/412C).

Subgroups of interest were defined based on the size and number of AMLs at the index date, measured as part of the regular care at the UMCU. Patients with the largest AML at the index date of ≥ 3.5 cm formed the subgroup of patients with large AMLs, while patients with the largest AML at the index date of < 3.5 cm formed the subgroup of patients with small AMLs [17, 18]. Patients with small AMLs were further divided into patients with ≤ 5 and > 5 small AMLs, but stratification was not possible in the large AML subgroup where only two patients had > 5 AMLs. The cutoffs for the size and number of AMLs correspond to the cutoffs between AML stages two and three in the AML staging criteria [17, 18] (Online Resource 1).

Treatments received included nephrectomies, embolizations, kidney transplants, and potential off-label mTORi. Disease monitoring was measured in terms of the use of diagnostic renal and non-renal scans (e.g., CT scans, ultrasounds), visits for sAML follow-up, and visits with specialists. Kidney function was based on glomerular filtration rate (eGFR) calculated from serum creatinine measurements. Characteristics of patients with sAML measured pre-index or at the index date are listed in Table 1. Hypertension was defined based on systolic and diastolic blood pressure measurements closest to the index date using NIH criteria [19]. Chronic kidney disease (CKD) stages were determined from eGFR levels [20, 21].

**Table 1:** Patient characteristics at index date, overall, and stratified by size and number of renal AMLs

	Patients with largest AML^a^ at index date ≥ 3.5 cm	Patients with largest AML^a^ at index date < 3.5 cm		
		All patients	Stratification by the number of AMLs	
			≤ 5 small AMLs	> 5 small AMLs
	(*N* = 26)	(*N* = 27)	(*N* = 21)	(*N* = 6)
Patient age (years), mean ± SD [median]	51.2 ± 12.7 [54.0]	52.0 ± 13.6 [55.1]	53.0 ± 14.3 [56.0]	48.4 ± 11.2 [45.4]
Female, *n* (%)	22 (84.6)	23 (85.2)	17 (81.0)	6 (100.0)
Caucasian race, *n* (%)	26 (100)	27 (100)	21 (100)	6 (100)
sAML stage^b^, *n* (%)				
0	0 (0.0)	5 (18.5)	5 (23.8)	0 (0.0)
1	0 (0.0)	14 (51.9)	13 (61.9)	1 (16.7)
2	1 (3.8)	7 (25.9)	2 (9.5)	5 (83.3)
3	19 (73.1)	1 (3.7)	1 (4.8)	0 (0.0)
4	4 (15.4)	0 (0.0)	0 (0.0)	0 (0.0)
5	1 (3.8)	0 (0.0)	0 (0.0)	0 (0.0)
6	1 (3.8)	0 (0.0)	0 (0.0)	0 (0.0)
Bilateral renal AMLs, *n* (%)	13 (50.0)	11 (40.7)	6 (28.6)	5 (83.3)
Prior sAML bleeding episodes, *n* (%)	8 (30.8)	1 (3.7)	0 (0.0)	1 (16.7)
eGFR (mL/min/1.73 m^2^) at index date^c^, *n* (%)				
≥ 90 (normal eGFR)	6 (23.1)	10 (37.0)	6 (28.6)	4 (66.7)
60–89 (CKD stage 2)	14 (53.8)	11 (40.7)	9 (42.9)	2 (33.3)
45–59 (CKD stage 3A)	2 (7.7)	3 (11.1)	3 (14.3)	0 (0.0)
30–44 (CKD stage 3B)	0 (0.0)	0 (0.0)	0 (0.0)	0 (0.0)
15–29 (CKD stage 4)	0 (0.0)	0 (0.0)	0 (0.0)	0 (0.0)
Under 15 (CKD stage 5)	0 (0.0)	0 (0.0)	0 (0.0)	0 (0.0)
Unknown	4 (15.4)	3 (11.1)	3 (14.3)	0 (0.0)
Prior nephrectomy, *n* (%)	2 (7.7)	4 (14.8)	3 (14.3)	1 (16.7)
Prior embolization, *n* (%)	2 (7.7)	0 (0.0)	0 (0.0)	0 (0.0)
Hypertension at index date^d^, *n* (%)				
Yes	9 (34.6)	4 (14.8)	3 (14.3)	1 (16.7)
No	4 (15.4)	5 (18.5)	3 (14.3)	2 (33.3)
Unknown	13 (50.0)	18 (66.7)	15 (71.4)	3 (50.0)
LAM, *n* (%)	4 (15.4)	3 (11.1)	2 (9.5)	1 (16.7)
AMLs in organs other than kidney or lung^e^, *n* (%)	2 (7.7)	0 (0.0)	0 (0.0)	0 (0.0)
History of pneumothorax, *n* (%)	2 (7.7)	1 (3.7)	1 (4.8)	0 (0.0)
History of pulmonary nodule, *n* (%)	2 (7.7)	2 (7.4)	2 (9.5)	0 (0.0)
Duration post-index follow-up^f^ (years), mean ± SD [median]	5.5 ± 4.5 [4.3]	3.5 ± 2.9 [3.1]	4.2 ± 3.0 [3.8]	1.2 ± 0.6 [1.1]
Observation period prior to sAML diagnosis^g^ (years), mean ± SD [median]	1.9 ± 4.2 [0.2]	3.6 ± 6.3 [0.6]	4.8 ± 7.2 [0.6]	0.8 ± 0.5 [1.0]

*sAML*, sporadic angiomyolipoma; *SD*, standard deviation; *LAM*, lymphangioleiomyomatosis; *eGFR*, estimated glomerular filtration rate

^a^As reported in the chart; the subgroup with ≤ 5 small AMLs includes one patient without any renal AMLs at index date (the patient had nephrectomy pre-index)

^b^As reported in the chart; please see Online Resource 1 for the renal AML staging criteria used in this study

^c^Reported as part of the sAML assessment for almost all patients

^d^Defined as systolic pressure ≥ 140 mmHg or diastolic pressure ≥ 90 mmHg (*source:* NIH MedlinePlus, http://www.nlm.nih.gov/medlineplus/magazine/issues/winter10/articles/winter10pg10a.html, accessed August 13, 2015). For patients with multiple blood pressure measurements within this period, hypertension status is based on the measurement closest to the index date within 90 days before or after the index date

^e^One patient had AMLs in the pancreas; another patient had AMLs in the liver

^f^From index date to date of the last recorded visit or date of data collection (April 2015), whichever came first

^g^From first recorded visit to sAML diagnosis

Rates of disease monitoring per patient per year (PPPY) were calculated as the number of scans/visits divided by the number of patient-years of follow-up and were compared between subgroups of interest using regressions with Poisson distribution. Mean eGFR levels were reported for several age groups such as 25 to 34 years and ≥ 65 years by averaging the eGFR test values of all patients observed during the respective age groups. Mean eGFR levels across different ages were presented for comparison purposes alongside the corresponding mean eGFR levels across ages previously reported for the general Dutch population [22, 23] and the TSC population [18]. At each age, the mean eGFR levels from the general Dutch population [22, 23] were standardized to the sex distribution of the AML study sample at that age.

## Results

### Patient characteristics

**Fig. 1:**
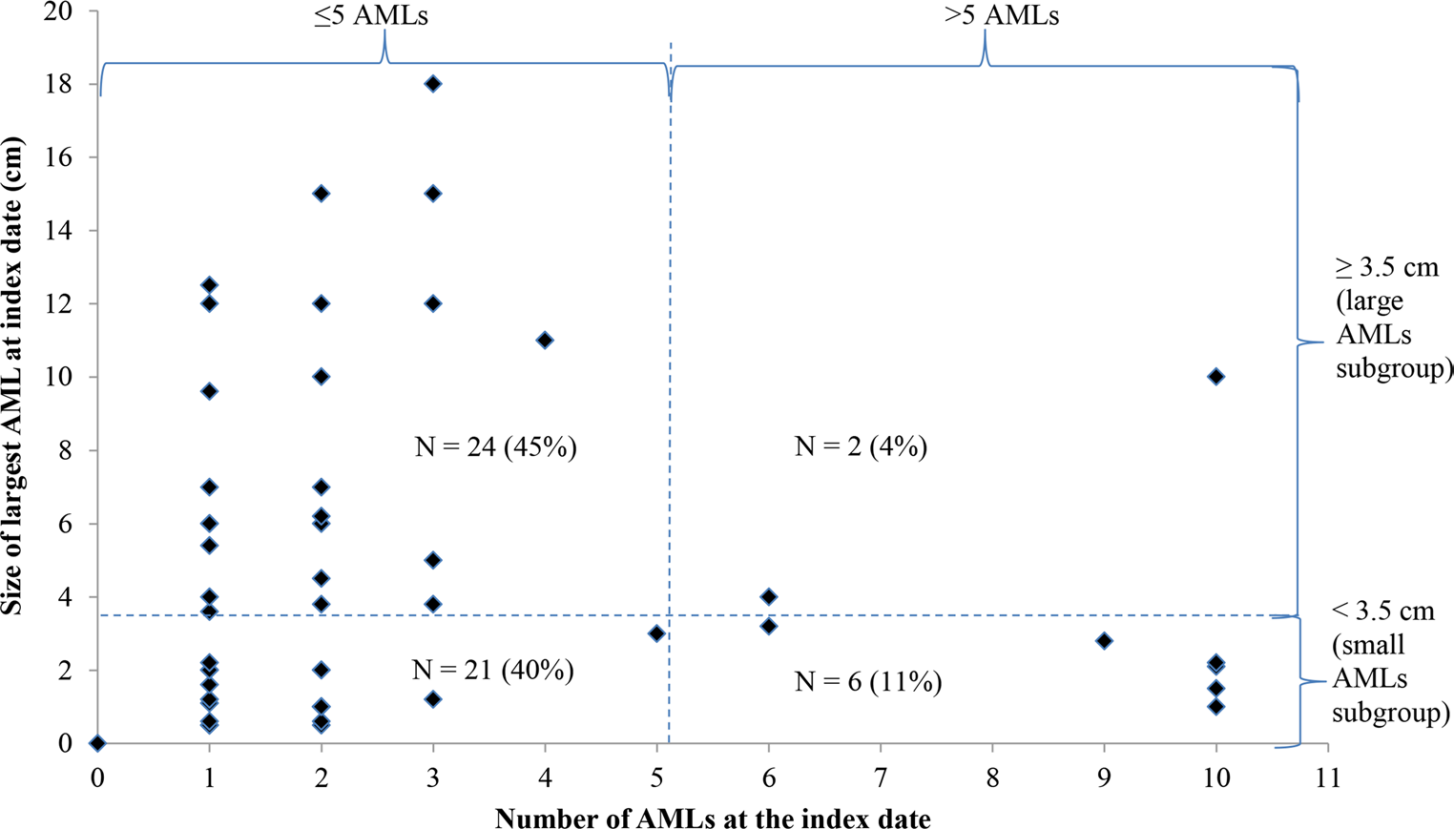
Number and size of AMLs at index date among the 53 patients in the sAML study sample

The study sample included 53 patients, followed for a total of 184.6 patient-years. The large and small sAML subgroups included 26 and 27 patients, respectively. Only six patients in the small sAML subgroup had > 5 AMLs, but they accounted for 75% of all patients with > 5 AMLs (Table 1, Fig. 1). The largest AML observed in the study sample was 18 cm (Fig. 1).

Median age at index date ranged from 48 to 53 years across subgroups (Table 1). Patients with large AMLs had the highest frequency for the history of sAML bleeding (31%) and hypertension (35%), but the lowest rate of pre-index nephrectomy (8%). No patient had CKD stage ≥ 3B at the index date. Bilateral renal AMLs were found in 83% of patients with > 5 small AMLs and 50% of patients with large AMLs. LAM was observed in 10–17% of the patients, depending on the group. AML in other organs was reported in two patients, both in the large AML subgroup.

### Treatments

**Fig. 2:**
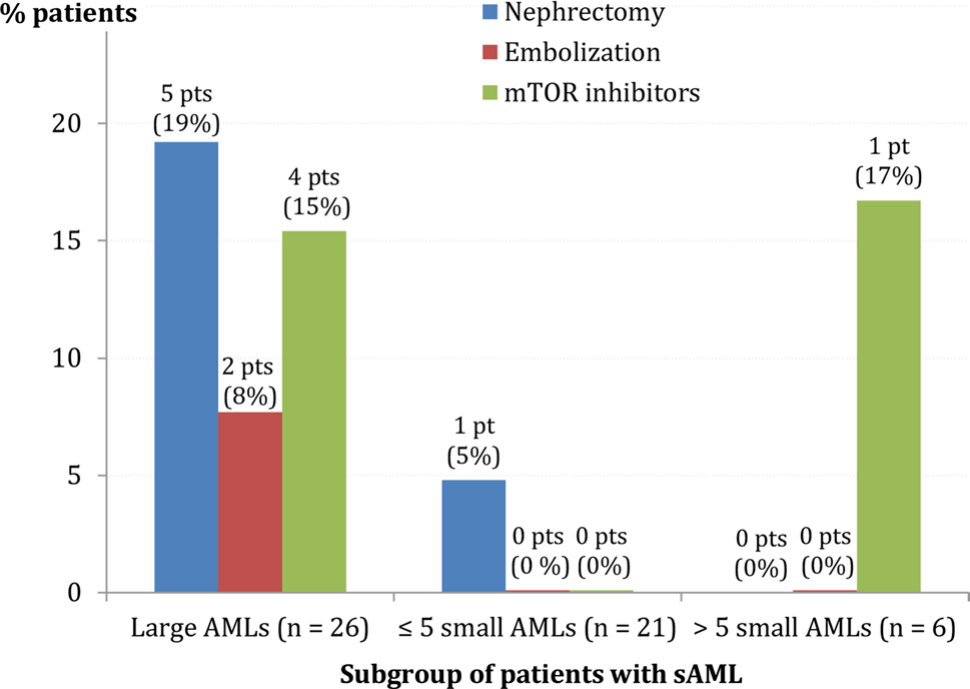
Treatments received during the follow-up period. *sAML*, sporadic angiomyolipoma; *mTOR inhibitor*, inhibitor of mammalian target of rapamycin; *pt*, patient

In the post-index period, six (11%) patients underwent a nephrectomy and two (4%) patients underwent an embolization. (One patient underwent both interventions.) These interventions appeared to be more common among patients with large AMLs than patients with small AMLs (19 vs. 4% and 8 vs. 0%, respectively; Fig. 2), and there were no kidney transplants. Off-label mTORi were received by five (9%) of patients (four [15%] and one [17%] patients with large AMLs and > 5 small AMLs, respectively), with three patients experiencing a decrease in AML size post-mTORi initiation and two patients experiencing no change in AML size (Online Resource 2).

### Disease monitoring

**Fig. 3:**
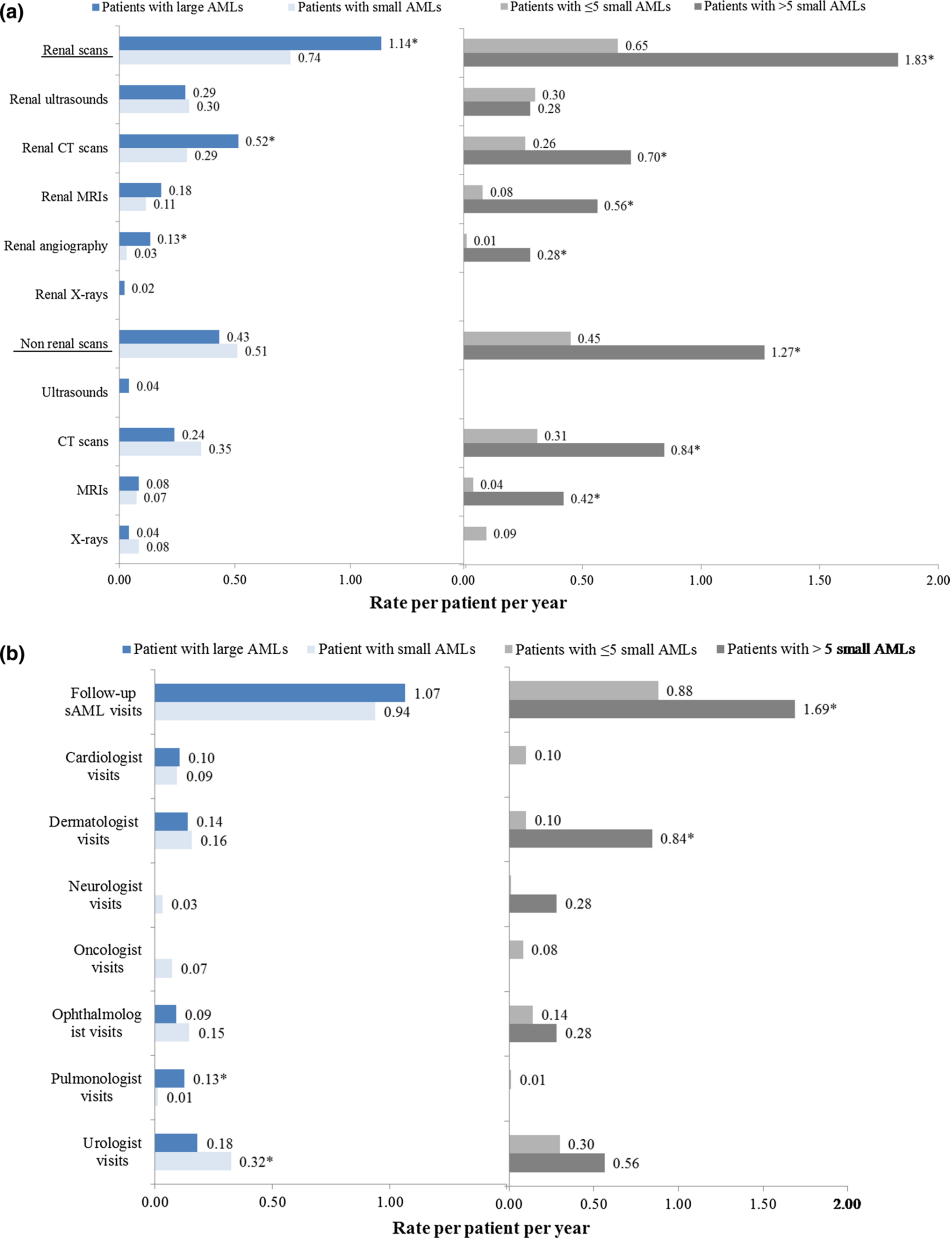
Disease monitoring in the follow-up period. Panel a Scans. Panel b Visits. *Rates statistically different between groups (*P* < 0.05)

Renal scan monitoring was highest for patients with > 5 small AMLs and lowest for patients with ≤ 5 small AMLs (Fig. 3a). Patients with large AMLs had significantly higher rates of renal scans compared with patients with small AMLs, but lower rates than the subgroup of patients with > 5 small AMLs. Patients with > 5 small AMLs used significantly more renal scans than patients with ≤ 5 small AMLs, with the exception of renal ultrasounds. Non-renal scan use, mainly CT scans and MRIs, was similar in patients with large and small AMLs, but significantly higher in patients with > 5 versus ≤ 5 small AMLs.

Frequency of sAML follow-up visits post-index was also highest for patients with > 5 small AMLs and lowest for patients with ≤ 5 small AMLs (Fig. 3b). There was a non-statistically significant trend toward higher rates of sAML follow-up visits among patients with large AMLs (1.07 visits PPPY) compared to patients with small AMLs (0.94 visits PPPY, P = 0.342). As compared to patients with ≤ 5 small AMLs, patients with > 5 small AMLs had significantly higher rates of visits to pulmonologists (0.13 vs. 0.01 visits PPPY, *P* = 0.016) and dermatologist (0.84 vs. 0.10 visits PPPY, *P* < 0.001) and lower rates of visits to urologist (0.18 vs. 0.32 visits PPPY, *P* = 0.029).

### Renal function

**Fig. 4:**
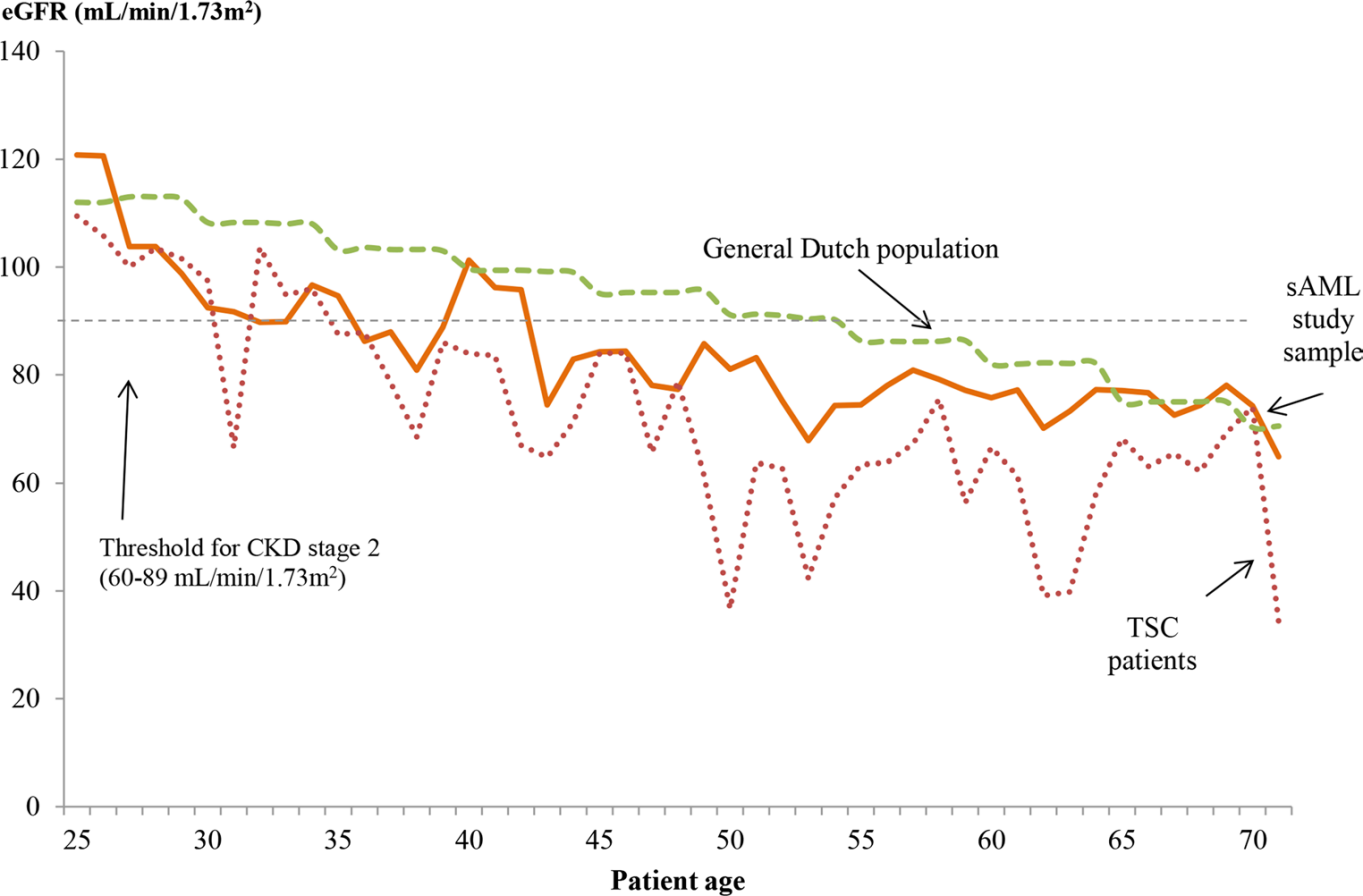
eGFR by age among patients in the sAML study sample^a^, patients with TSC^b^, and the general Dutch population^c^. *eGFR*, estimated glomerular filtration rate; *sAML*, sporadic angiomyolipoma; *TSC*, tuberous sclerosis complex. ^a^A patient may contribute multiple times to a given age category and to multiple age categories. ^b^Data points by age extracted from Vekeman et al. JME 2015. ^c^Data points by age group extracted from Wetzels JFM et al. Kidney International 2007 standardized to the sex distribution in the sAML study sample

As compared to patients with small AMLs, the mean eGFR of patients from the sAML study sample with large AMLs was lower at young ages (87.0 vs. 108.1 mL/min/1.73 m^2^/year at the age of 25–34 years; 81.1 vs. 100.8 at 35–44 years; 77.0 vs. 92.8 at 45–54 years) and higher at older ages (77.4 vs. 60.8 at ≥ 65 years; Table 2). Patients with sAML developed CKD stage two at a younger age on average compared to patients in the general Dutch population [23] (age 42 vs. 55 years), but later than the patients with TSC (age 42 vs. 35 years; Fig. 4) [18]. Patients on average had 5.9 eGFR measurements over the follow-up period. Among patients with 10 or more eGFR measurements (*N* = 6), the trends were mixed (Online Resource 3).

**Table 2:** eGFR by age group and size of AMLs

	Mean eGFR (mL/min/1.73 m^2^)				
	Age 25–34^a^	Age 35–44^a^	Age 45–54^a^	Age 55–64^a^	Age ≥ 65^a^
All patients with sAML	98.9 (6 pts)	86.8 (14 pts)	80.3 (13 pts)	75.6 (24 pts)	67.3 (14 pts)
Large AMLs subgroup	87.0 (3 pts)	81.1 (8 pts)	77.0 (7 pts)	74.0 (14 pts)	77.4 (8 pts)
Small AMLs subgroup^b^	108.1 (3 pts)	100.8 (6 pts)	92.8 (6 pts)	78.2 (10 pts)	60.8 (6 pts)

*eGFR*, estimated glomerular filtration rate; *AML*, angiomyolipoma; *pts*, patients

^a^One patient may have contributed multiple times to a given age category and to multiple age categories

^b^Age group stratification was not possible for the subgroups of patients with ≤ 5 small AMLs and > 5 small AMLs due to the small number of patients

## Discussion

This study investigated the natural history and treatment of 53 patients with sAML followed in a large tertiary center in the Netherlands, overall and stratified by the size and number of AMLs. Large AMLs were common in this population, posed a high burden on the patient in the form of comorbidities and invasive interventions and required closer monitoring than small AMLs. The 25% of the patients with small AMLs who had > 5 small AMLs had the highest rates in the sAML population for renal scans and follow-up sAML visits. The results from the study also support the hypothesis that AMLs impact kidney function, as indicated by the earlier onset of renal impairment in both patients with sAML and TSC as compared to the general population.

The literature on sAML is scarce, with most previous studies relying on small case series or case series aggregated data, often without distinguishing between patients with sAML and patients with TSC-AML [3, 6, 8, 11]. With a median age of 54 years and a predominance of females, patients in the current study have a similar age and sex distribution to patients with AMLs in previous studies [6, 11, 24, 25]. However, advanced sAML may have been more common at the index date in the current study as compared to other studies: 43% patients had tumors > 4 cm [data not shown] versus 14–22% in other studies [9, 25], 36% had a single AML versus 77–80% [11, 25] and 45% had bilateral AML versus 5–16% [9, 11]. Consistent with this finding, more patients in the current study underwent a nephrectomy or embolization during their lifetime (26%) than patients in other contemporary studies (range 5.6–13%) [7, 11]. These intervention rates are nevertheless lower than the rates observed in older studies [3, 10], reflecting a possible shift in sAML disease management, where active surveillance is considered a safer practice, and better imaging techniques reduce the unnecessary nephrectomy related to misinterpretation of the AML as a renal carcinoma [11].

As expected based on current guidelines [14], nephrectomy and embolization were performed mainly on patients with large AMLs. Other studies have also showed that 86–100% of all interventions were in patients with large AMLs [6, 9]. Interestingly, more patients with sAML underwent nephrectomy than embolization than was previously reported for TSC-AML [17, 26]. This may be due to the aforementioned possible shift in clinical practice if the sAML and TSC-AML patients from the two studies reflected different treatment eras, but it is also possible that nephrectomies may be considered more risky interventions in TSC-AML. Five patients (9.4%) in the current sample received treatment with mTORi, including four with large AMLs (range: 7–18 cm) and four with CKD at the time of treatment initiation (eGFR range: 65–85). Interestingly, all three patients with CKD who had eGFR measurements before and after mTORi initiation had an improvement in eGFR after mTORi initiation (range: 8–15% eGFR increase; data not shown). These results suggest that mTORi could play a role in these patients, as previously hypothesized based on the presence of inappropriately regulated mTOR pathway in sAML [16].

The current study also found that rates of renal scans and rates of follow-up visits for sAML were higher among patients with large AMLs and patients with > 5 small AMLs. The finding that patients with large AMLs are monitored closely is in line with current recommendations for patients known to be at an increased risk of complications such as spontaneous hemorrhage or flank pain [26, 27]. While patients with small AMLs were not traditionally considered to be high-risk patients, recent studies showing that AMLs may grow faster when multiple tumors are present [28] may have changed the risk assessment for these patients. However, it is also possible that sAML differential diagnosis is more difficult in patients with small AMLs, thus requiring more testing to potentially rule out TSC or malignant neoplasms. The study finding that patients with > 5 small AMLs also had high rates of non-renal scans and visits to dermatologist and ophthalmologists suggests that at least some of these patients undergo testing for TSC.

This study has a number of limitations. First, results cannot be generalized to the entire sAML population and reflect the patients followed in specialized tertiary centers in the Netherlands. Second, differences in renal function between patients at different age groups should not be interpreted as rates of decline by age in renal function because different patients may have contributed to different age groups. Third, part of the kidney function decline in patients with nephrectomy prior to the index date may be related to the prior nephrectomy. Given that patients with small AMLs had higher frequency of prior nephrectomies, the eGFR gap observed between the large and small AML subgroups may have been underestimated at younger ages and overestimated at older ages. Fourth, information on treatment received outside the UMCU was limited to the physician notes and may be subject to missing information. However, all major interventions and key events that occurred outside the UMCU were likely captured in the patients’ medical history. Fifth, imaging tests have become in recent years more sensitive to detect small AMLs. This is consistent with the study finding that patients with > 5 small AMLs had shorter follow-up (i.e., were diagnosed as such more recently) than patients with ≤ 5 small AMLs. The true proportion of patients with > 5 small AMLs may be underestimated.

In conclusion, large AMLs are common among patients with sAML and pose a high burden on the patient in the form of comorbidities and invasive interventions. As compared to patients with small AMLs, patients with large AMLs were more likely to have bilateral renal AMLs, LAM, hypertension, AML bleeding at the study onset; appeared to be at an increased risk of undergoing a nephrectomy, embolization, and having early onset of kidney impairment; and had higher rates of renal scans. While the subgroup of patients with > 5 small AMLs included only six patients, most of these patients had bilateral renal AMLs and some patients also had a history of bleeding and nephrectomy, suggesting this subgroup may also pose a high comorbidity and interventional burden. Furthermore, these patients had high rates of renal scans and follow-up sAML visits as compared to patients with ≤ 5 small AMLs. Due to the potential of sAMLs to grow over time and the detrimental and costly long-term clinical outcomes for patients with large and/or multiple AMLs, there is an urgent need for therapies targeting a possible mTOR pathway in AML and for interventions that can prevent or delay the growth of AMLs without impairing the kidney.

## Electronic supplementary material

Below is the link to the electronic supplementary material.

eFig. 1 Change of eGFR over time among patients in the sAML study sample for whom there was sufficient eGFR information (N=6) (DOCX 50 kb)
