# Supplementary material for: Factors associated with the number and size of renal angiomyolipomas in sporadic angiomyolipoma (sAML): a study of adult patients with sAML managed in a Dutch tertiary referral center
Source: Int Urol Nephrol. 2018 Jan 15;50(3):459–67. doi: 10.1007/s11255-017-1766-9 (PMC5845070; doi:10.1007/s11255-017-1766-9)
Supplement: Supplementary file 1 — eFig. 1 Change of eGFR over time among patients in the sAML study sample for whom there was sufficient eGFR information (N=6) (DOCX 50 kb) [file 11255_2017_1766_MOESM1_ESM.docx]

**ONLINE SUPPLEMENTAL MATERIAL**

Factors associated with the number and size of renal angiomyolipomas in sporadic angiomyolipoma (sAML): a study of adult patients with sAML managed in a Dutch tertiary referral center

International Urology and Nephrology

JLH Ruud Bosch, Francis Vekeman, Mei Sheng Duh, Maureen Neary, Matthew Magestro, Jonathan Fortier, Paul Karner, Raluca Ionescu-Ittu, Bernard A. Zonnenberg (Department of Internal Medicine, University Medical Center Utrecht, [B.Zonnenberg@kpnmail.nl](mailto:B.Zonnenberg@kpnmail.nl))

**Online Resource 1.** Renal angiomyolipoma staging criteria

| **Stage^a^** | **No. of AMLs** | **AML size** | **Kidney anatomy** |
| --- | --- | --- | --- |
| 0 | None^b^ | | Normal |
| 1 | ≤5 | 1 cm to 3.5 cm | Normal |
| 2 | ˃5 | 1 cm to 3.5 cm | Normal |
| 3 | ≤5 | At least 1 AML ≥3.5 cm | Kidney intact |
| 4 | ˃5 | 1 to 4 AMLs ≥3.5 cm | Kidney intact |
| 5 | ˃5 | 5 or more AMLs ≥3.5 cm | Kidney recognizable |
| 6 | ˃5 | At least 1 AML ≥5.0 cm | Kidney not recognizable |

a. All three conditions need to be satisfied.

b. Not able to definitely determine via CT scan if lesions < 1 cm in longest diameter are angiomyolipomata.

**Online Resource 2.** Response to treatment in patients treated with mTOR inhibitors

| **Patient** | **mTORi Treatment (Months of Use)** | **Response to treatment (% decrease AML size since mTORi initiation)** |
| --- | --- | --- |
| A | everolimus (13 months) | 43% |
| B | everolimus (3 months) | 0% |
| C | sirolimus (78 month) followed by everolimus (12 months) | 32% |
| D | sirolimus (12 months) | 12% |
| F | everolimus (42 months) | 0% |

**Online Resource 3.** eGFR over time among patients with ≥10 measurements
